# Effect of moxibustion on quality of life after chemotherapy in patients with the malignant tumor: A protocol for systematic review and meta analysis

**DOI:** 10.1097/MD.0000000000023471

**Published:** 2021-01-22

**Authors:** Dan Tao, Jingyu Xu, Shuyuan Zou, Yanfu Tan, Shuangchun Ai

**Affiliations:** a Chengdu University of Traditional Chinese Medicine, Chengdu, Sichuan Province; b First Teaching Hospital of Tianjin University of Traditional Chinese Medicine; c National Clinical Research Center for Chinese Medicine Acupuncture and Moxibustion; d Tianjin University of Traditional Chinese Medicine, Tianjin; e Mianyang Hospital of T.C.M, Mianyang, Sichuan Province, China.

**Keywords:** malignant tumor, meta-analysis, moxibustion, protocol, quality of life

## Abstract

**Background:**

The quality of life of patients with malignant tumor can be affected by the severity and treatment of the disease. After chemotherapy, the main symptoms are gastrointestinal reactions, including nausea, vomiting, loss of appetite, and so on, and hematologic response including leukopenia, anemia, and bleeding in severe cases. Currently, moxibustion is conducted to improve the living life of patients after chemotherapy. This article will make a comprehensive and objective discussion in terms of the effect of moxibustion on quality of life after chemotherapy in patients with malignant tumors.

**Methods:**

After searching the Chinese database (CNKI, VIP, Wanfang Database, and Chinese Biomedical Database) and English database (PubMed, Embase, the Cochrane Library Web of Science), Meta-analysis was performed according to the randomized controlled trial of moxibustion after chemotherapy in patients with malignant tumors. The retrieval time was limited from the time of building the repository to October 2020. Two researchers independently conducted data extraction and quality evaluation of literature on the included studies, and RevMan5.3 was used for Meta-analysis on the included literature.

**Results:**

After analyzing the included literature, this study suggested that by combining chemotherapy with moxibustion, the adverse reactions such as nausea, vomiting, appetite, and insomnia can be relieved. Meanwhile, the psychological burden of patients can be alleviated to a certain extent. Therefore, moxibustion can improve the overall health level and quality of life of patients with malignant tumors.

**Conclusion:**

This study will provide evidence-based medical evidence that moxibustion can improve the quality of life after chemotherapy and reduce chemotherapy's adverse reactions in patients with malignant tumors.

**Ethics and dissemination:**

Private information from individuals will not be published. This systematic review also does not involve endangering participant rights. Ethical approval was not required. The results may be published in a peer-reviewed journal or disseminated at relevant conferences.

**OSF Registration number:**

DOI 10.17605/OSF.IO/Q5NYM.

## 1 Introduction

The malignant tumor is a disease caused by excessive proliferation of cells which losing normal regulation in the body. It is also known as cancer which is known as one of the three unnatural death diseases. Its morbidity and mortality are rising year by year in the world.^[1]^ And chemotherapy is currently the main and the most effective ways of treating cancer, the characteristics of its systemic therapy control the proliferation of tumor cells effectively, and prolong life for patients with tumors, but the side effects of cytotoxic drugs will cause digestive system reaction and organ harm might influence the quality of life and late nursing of patients.^[2]^

According to traditional Chinese medicine theory, the invasion of poison is the key factor to cause the pathogenesis of malignant tumor, and the internal factor is insufficient anti-pathogenic energy.^[3]^ Therefore, cancer treatment in Chinese medicine mainly focuses on strengthening the body resistance to eliminate pathogenic factors, and it will adjust according to specific conditions. Moxibustion is a therapeutic method by burning moxa with the shape of strips or columns put in the corresponding parts for fumigation and burning. It mainly relies on the heating power generated by burning to warm the skin then acts on the acupoints or meridians to play a part in the warm channel and expelling cold, supporting the healthy atmosphere, and promoting qi to activate blood.^[4]^ Because of its simpler operation and comfortable process, it is accepted by people step by step.

Moxibustion therapy has been used in the treatment of tumors in clinical practice for many years and the related mechanism researches have achieved a lot of achievements.^[5]^ At present, modern experimental studies have shown that traditional moxibustion can enhance the immunity of patients. By increasing the number of peripheral blood leukocyte, it adjusts the functions of systems of the body and enhances the specific and non-specific immune functions, so as to regulate the immune function of the body well.^[6]^ Clinical studies have shown that moxibustion can alleviate a series of side effects after the surgery of malignant tumor, radiotherapy and chemotherapy, so as to reduce the clinical symptoms.^[7]^ However, there are some differences among clinical trials in terms of study design, efficacy and other aspects, and the analysis is not comprehensive.^[8]^ Therefore, the reliability of results can be influenced to a certain extent. This study conducted the Meta-analysis by searching the randomized controlled trials (RCTs) of moxibustion intervention in patients with malignant tumors after chemotherapy in Chinese and English databases.

## 2 Methods

### 2.1 Protocol register

This systematic review protocol and meta-analysis have been drafted under the guidance of the preferred reporting items for systematic reviews and meta-analysis protocols (PRISMA-P). In addition, it has been registered on the open science framework (OSF) on October 28, 2020 (registration number: DOI 10.17605/OSF.IO/Q5NYM).

### 2.2 Ethics

This study is a document analysis without patients’ personal information and privacy. Therefore, there is no need to get approval from the Ethics Committee and acquire informed consent of patients.

### 2.3 Eligibility criteria

#### 2.3.1 Types of studies

We collected all available RCTs on moxibustion's effects on quality of life in patients with malignant tumors, regardless of blinding, publication status, or region, but the language is limited to Chinese and English.

#### 2.3.2 Objects of study

Malignant tumor patients who had received chemotherapy and had not received radiation therapy or surgery. There was not limited in age, gender, country, ethnicity, and tumor type. Patients should have clear awareness and volunteer to accept moxibustion therapy and cooperate actively.

#### 2.3.3 Intervening measure

Control group: Treatment was performed on the basis of Guidelines for Tumor Drug Administration. Both western medicine and proprietary Chinese medicine were available. There is no limitation of the type, dosage and course of these two kinds of medicines.

Treatment group: Based on the control group, moxibustion therapy (including direct contact moxibustion, ginger moxibustion, moxibustion with seed-sized moxa cone, blistering moxibustion, and mild moxibustion) was added, there is no limitation on the type of moxibustion, the acupuncture point, course of treatment, the time of moxibustion, and number of cones.

#### 2.3.4 Outcome indicator

1. Primary outcome: overall efficiency^[9]^;
2. Secondary outcome: symptoms of improvement, life quality score.

### 2.4 Exclusion criteria

1. Repeatedly published papers and studies with the complete data and the best quality of included literature;
2. The published literature is abstract or the data record is incomplete, and the complete data cannot be obtained after contacting the author;
3. Literature with obvious errors;
4. Review or literature research, animal experimental research;
5. The treatment group adopted other traditional Chinese therapy except for moxibustion, such as auricular point, acupuncture, and massage.

### 2.5 Search strategy

Using “moxibustion therapy,” “moxa-moxibustion,” “malignant tumor,” and “cancer” as Chinese index words, the literature search was conducted in CNKI, VIP, Wanfang Database, and CBM. Then using “moxibustion,” “moxa-moxibustion,” “cancer,” “tumor” as English index words, the literature search was conducted in English literature. The retrieval time was from the establishment of the database to October 2020, and all domestic and foreign RCTs on the effect of moxibustion on the quality of life of patients with malignant tumors were collected. Take PubMed as an example, and the retrieval strategy is shown in Table 1. All the domestic and foreign RCTs on the effect of moxibustion on the quality of life of malignant tumor patients were collected from the time of database construction to October 2020.

**Table 1:** Retrieval strategy of PubMed.

Number	Search terms
#1	Moxibustion[MeSH]
#2	Moxibustion[Title/Abstract]
#3	Moxa- moxibustion[Title/Abstract]
#4	Moxibustion treatment[Title/Abstract]
#5	#1 OR #2 OR #3 OR #4
#6	Neoplasms[MeSH]
#7	Neoplasm[Title/Abstract]
#8	Cancer[Title/Abstract]
#9	Tumor[Title/Abstract]
#10	Carcinoma[Title/Abstract]
#11	#6 OR #7 OR #8 OR #9 OR #10
#12	#5 AND #11

### 2.6 Data filtering and extraction

We used Cochrane Collaborative Reviewers’ Handbook 5.0 as the method. Using PRISMA flowcharts, EndNote X7 document management software independently filtered and verified documents’ compliance with the above inclusion and exclusion criteria. Finally, we checked the undetermined literature and had a discussion with a third researcher. Meanwhile, Excel 2013 software was used to extract relevant information, including

1. Clinical features (title, first author, year of publication, month, sample size, sex ratio, average age, and average course of the disease);
2. Intervening measure: moxibustion methods, number of strong moxibustion, acupuncture points and course of treatment, types of western medicine and Chinese patent medicine, method and frequency of administration and course of treatment in the treatment group; Name of western medicine and Chinese patent medicine, administration method, frequency of administration and course of treatment in the control group; Risk preferences for factors of evaluation in a randomized controlled study; Observed indicators.

**Figure 1:**
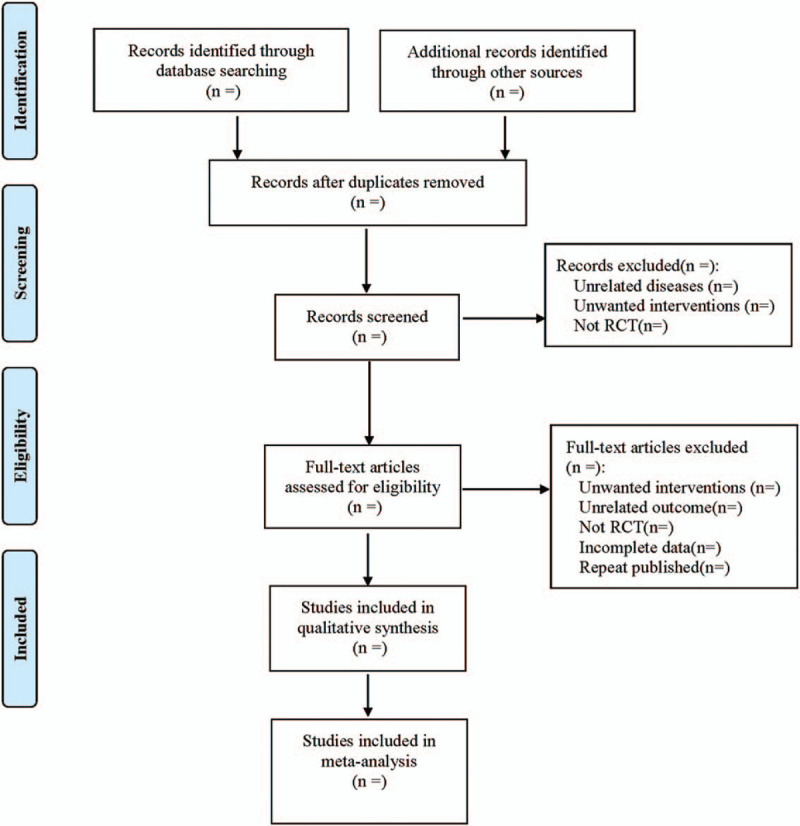
The process of literature filtering.

The process of literature filtering is shown in Figure 1.

### 2.7 Literature quality evaluation

We used the Cochrane Collaboration's tool for assessing the risk of bias to conduct the methodological quality evaluation of included studies, the content included:

1. Whether the stochastic approach is reasonable (low risk, ambiguity, and high risk).
2. Whether hidden allocation or not (low risk, ambiguity, and high risk).
3. Whether the blind method is reasonable, including single-blind and double-blind (low risk, ambiguity, and high risk).
4. Whether the bias of incomplete data (such as loss of follow-up or withdrawal, number of lost cases, reasons for lost cases) and selective reporting and other bias exist.

According to the literature's performance in the above evaluation items, two researchers determined the literature with three risk levels: low, middle, and high. If there is any dispute over literature, the definition should be discussed. According to the final number of literature, publication bias should be conducted after Egger's test.

### 2.8 Statistical analysis

#### 2.8.1 Data analysis and processing

We used RevMan5.3 software provided by Cochrane to analyze the data. If the included indicators are dichotomous variables, relative risk (RR) or odds ratio (OR) and its 95% confidence interval (CI) will be effect size. If included research indicators are continuous variables, weight mean difference (WMD) or standardized mean difference (SMD) with its 95%CI expression. And heterogeneity analysis of included study was conducted. Fixed-effect models will be used for meta-analysis when the heterogeneity between studies is low (*I*^2^ ≤ 50%); when heterogeneity is high among studies (*I*^2^ ≥ 50%), random effects model was used for Meta analysis, and descriptive analysis was used for incomplete data.

#### 2.8.2 Missing data processing

If the data is missing or incomplete, we will contact the corresponding author for the missing data. Otherwise, this study will be deleted.

#### 2.8.3 Subgroup analysis

Subgroup analysis was conducted according to the type of specific moxibustion therapy in treatment group; subgroup analysis was conducted according to the tumor type of patients; subgroup analysis was conducted according to patients’ age, and there were three groups which were children, teenagers, and old people, respectively; subgroup analysis was conducted according to the course of treatment.

#### 2.8.4 Sensitivity analysis

In order to test the stability of meta-analysis results of outcomes, a one-by-one elimination method will be adopted for sensitivity analysis.

#### 2.8.5 Assessment of reporting biases

For the major outcome indicators, if the included study was ≥10, a funnel plot was used to detect publication bias qualitatively. Egger's and Begg's tests are used to assess potential publication bias quantitatively.

#### 2.8.6 Evidence quality evaluation

The Grading of Recommendations Assessment, Development, and Evaluation (GRADE) will be used to assess the quality of evidence. It contains 5 domains (bias risk, consistency, directness, precision, and publication bias). And the quality of evidence will be rated as high, moderate, low, and very low.

## 3 Discussion

When the term “quality of life” was introduced into the medical research field, it is mainly referred to as the individual condition assessment of physical, psychological, and social functions, namely the health quality. Like survival and other types of clinical results, the patient's quality of life is an important index of accepting the effectiveness of health care services. With the improvement of living standards and the change of people's concept of life, this index has been paid more attention by more people.^[10]^ For malignant tumor patients, good quality of life guarantees to alleviate pain and prolonging life.

With a large population, our country has millions of patients suffering from malignant tumors every year. While focusing scientific research on cancer prevention, deepening research on cancer treatment cannot be ignored. Prevention and treatment of cancer with traditional Chinese medicine has made some achievements on the path of evidence-based development in the past 50 years. We have objectively proved the scientificity of ideas, such as strengthening the body resistance to eliminate pathogenic factors and prevention before disease onset by using evidence-based medical research methods. We also proved a series of effective and safe treatment methods.^[11]^ By analyzing the etiology and pathogenesis of tumors, methods and prescriptions can be determined by way of syndrome differentiation and treatment in Chinese medicine. The outstanding advantage of this way is overall intervention. Chinese medicine prescription and Chinese medicine have various effective constituents targeting pathogenesis of tumors. It can also improve the uncomfortable symptoms such as eating and sleeping.^[12]^ With the development of the times, acupuncture therapy has developed into other methods of treatment such as acusector, while it still follows the treatment principle of Chinese medicine at the same time as innovation. During the treatment of tumor, it shows the advantage of individualization and improve the side effects and quality of life after chemotherapy.^[13]^ It is widely known as excellent performance on acupuncture treating other diseases clinically.

The damage of immunocompetence of patients after chemotherapy belongs to consumptive disease in the traditional Chinese medicine. As a traditional therapy guided by the basic theory of Chinese medicine, moxibustion therapy follows the principle of treating deficiency with tonification. The commonly used acupuncture points of treatment of malignant tumors by Moxibustion are: Zusanli, Sanyinjiao, Dazhui. Zusanli, Sanyinjiao, and other points can regulate the spleen and stomach to consolidate basis. Dazhui acupoint can warm yang and benefit qi and strengthen the body resistance to eliminate pathogenic factors. Moxibustion activates the acupoints, promotes the movement of qi and blood, and regulates the nerve-endocrine-immune and zang-fu functions by hyperthermia.^[14]^ At present, clinical study on mechanism of moxibustion in tumor is also the mainly in the immune function. Studies show that moxibustion can participate in immune regulation.

IL-12, also known as natural killer cell stimulating factor (NKSF) and cytotoxic toxic lymphocyte maturation factor (CTMF), can promote the differentiation of Th0 cells into Th1 cells, and promote the maturation of natural killer cells (NK cells), thus forming the amplification effect of immunological enhancement. As an important bridge, it plays an important role in connecting the adaptive immunity of early non-specific innateimmunity and the laterantigen-specificity. Binds to TNF-α receptors on cell membranes of tumor, TNF-αcan killtumor cells specifically. Therefore, increased content of it may promote bleeding and necrosis of tumor cell, thereby inhibiting tumor growth. During the course of treatment, moxibustion can continuously increase the content of IL-12 and TNF-α, thereby improving the injury of immune function caused by chemotherapy.^[15]^ Using moxibustion on zusanli point can also effectively regulate the immunologic balance, relieve the clinical symptoms of patients. It has a certain therapeutic effect.^[16]^

At the beginning, the tumor is a local disease, but in the later period, it is essentially closely related to the functions of the systemic Zang-fu functions. Moxibustion therapy can alleviate the clinical symptoms of the patients with tumor from both local and overall aspects, and it has the effect of improvement on the quality of life of the patients.^[17]^ Moreover, the manipulation of moxibustion therapy is relatively simple and highly safe. Also, its price is relatively economical and it has no side effects. All of these advantages make it more and more accepted and selected by patients, so it is worthy of clinical application. Evidence-based medical research can provide reliable medical evidence for moxibustion to improve the quality of life of patients with malignant tumor so as to help the promotion of moxibustion. Therefore, moxibustion can be adopted by more clinicians.

The included literature in this study has the risk of bias, especially the generation of random sequence and the specific implementation of blind method. Other aspects, such as allocation and concealment, have not been mentioned the risk of bias, which indicates that the risk of bias may large in methodology, and also reflects that there are deficiencies in the design of RCTs in some literatures. Insufficient allocation, concealment and low use of blind method will seriously affect the authenticity of the conclusion, so as to reduce the reliability of the research results. Meanwhile, the included literatures only contain the literatures in Chinese and English, which may ignore the literatures in other languages that meet the requirements, so there is a certain publication bias.

### 3.1 Uncited references

^[18–21]^.

## Author contributions

**Data curation:** Dan Tao, Jingyu Xu.

**Funding acquisition:** Shuangchun Ai.

**Software:** Shuyuan Zou.

**Writing – original draft:** Dan Tao, Jingyu Xu.

**Writing – review & editing:** Yanfu Tan, Shuangchun Ai.
